# The Saprophytic Lifestyle of *Listeria monocytogenes* and Entry Into the Food-Processing Environment

**DOI:** 10.3389/fmicb.2022.789801

**Published:** 2022-03-08

**Authors:** Antonio Lourenco, Kristina Linke, Martin Wagner, Beatrix Stessl

**Affiliations:** ^1^Department of Food Biosciences, Teagasc Food Research Centre, Co. Cork, Ireland; ^2^Unit for Food Microbiology, Institute for Food Safety, Food Technology and Veterinary Public Health, University of Veterinary Medicine, Vienna, Austria; ^3^Austrian Competence Center for Feed and Food Quality, Safety and Innovation, Tulln, Austria

**Keywords:** *Listeria monocytogenes*, saprophyte, environment, genotypes, soil, water

## Abstract

*Listeria monocytogenes* is an environmentally adapted saprophyte that can change into a human and animal bacterial pathogen with zoonotic potential through several regulatory systems. In this review, the focus is on the occurrence of *Listeria sensu stricto* and *sensu lato* in different ecological niches, the detection methods, and their analytical limitations. It also highlights the occurrence of *L. monocytogenes* genotypes in the environment (soil, water, and wildlife), reflects on the molecular determinants of *L. monocytogenes* for the saprophytic lifestyle and the potential for antibiotic resistance. In particular, the strain-specific properties with which some genotypes circulate in wastewater, surface water, soil, wildlife, and agricultural environments are of particular interest for the continuously updating risk analysis.

## The Genus *Listeria*

*Listeria* spp. are Gram-positive, facultative anaerobic, non-spore forming, and catalase positive rods (Rocourt and Buchrieser, 2007). The genus currently includes 28 species and six subspecies with taxonomic names effectively published under the rules of the international code of nomenclature of bacteria. Orsi and co-workers separated the species into two phenotypically and genotypically distinct groups: *Listeria sensu lato* and *Listeria sensu stricto* (Orsi and Wiedmann, 2016). The first group is comprised of a pathogenic saprophytes present in the environment and includes *L. grayi* (subsp. grayi, and murrayi; Rocourt et al., 1992), *L. rocourtiae* (Leclercq et al., 2010), *L. fleischmannii* (subsp. fleischmannii and coloradonensis; Bertsch et al., 2013; den Bakker et al., 2013), *L. weihenstephanensis* (Halter et al., 2013), *L. floridensis*, *L. aquatica*, *L. cornellensis*, *L. riparia*, *L. grandensis* (den Bakker et al., 2014), *L. booriae*, *L. newyorkensis* (Weller et al., 2015a), *L. costaricensis* (Núñez-Montero et al., 2018), *L. goaensis* (Doijad et al., 2018), *L. thailandensis* (Leclercq et al., 2019), *L. valentina* (Quereda et al., 2020), *L. portnoyi*, and *L. rustica* (Carlin et al., 2021a).

The *Listeria sensu stricto* group includes the species *L. innocua* (Seeliger, 1981), *L. welshimeri* (Rocourt and Grimont, 1983), *L. seeligeri* (Rocourt and Grimont, 1983), *L. marthii* (Graves et al., 2010), *L. farberi*, *L. immobilis*, *L. cossartiae* (subsp. cossartiae and cayugensis; Carlin et al., 2021a,b), and the two pathogenic *Listeria* species *L. monocytogenes* (Murray et al., 1926; Pirie, 1940) and *L. ivanovii* (subsp. *ivanovii* and *londoniensis*; Seeliger et al., 1984; Boerlin et al., 1992). These two species are known to be pathogenic for both humans and animals (biohazard level 2), nevertheless *L. ivanovii* has only been associated with human disease very rarely (Guillet et al., 2010).

Generally, *Listeria sensu stricto* are able to grow at low temperatures (<4°C) and are motile due to the presence of flagella (Walker et al., 1990; Orsi and Wiedmann, 2016). Fully formed flagella are usually observed at temperatures between 20°C and 28°C since flagellin expression, required for assembly of flagella, is temperature dependent and reduced at 37°C (Peel et al., 1988). The regulation of this expression has been shown to be different between *L. innocua* and *L. monocytogenes*. *Listeria innocua* is motile at 37°C; however, only extremely rarely isolates of *L. monocytogenes* show reduced repression of flagellin production with consequent maintenance of motility at this temperature (Kathariou et al., 1995).

*L. monocytogenes* is known to cause both sporadic or epidemic listeriosis in high-risk groups (elderly, neonates, pregnant women, and immunocompromised people) and its incidence has been steadily increasing in the EU/EEA. There are two courses of listeriosis: a non-invasive and an invasive form. Non-invasive listeriosis (febrile gastroenteritis) is a mild form of the disease that mainly affects healthy people. The hospitalization rate for the invasive form of listeriosis is 95% and includes symptoms as sepsis, meningitis, rhomboencephalitis, and abortion (Allerberger and Wagner, 2010; EFSA and ECDC, 2019). Listeriosis is one of the most serious foodborne diseases under surveillance as it may lead to high morbidity and mortality rates in high-risk groups (20–30%; Watson, 2009; Allerberger and Wagner, 2010; EFSA and ECDC, 2015; EFSA, 2018). The transmission to consumers often occurs *via* contaminated food (Wesley, 2007). This review provides an overview of the current knowledge on the lifestyle of the genus *Listeria* in the environment addressing particular factors relevant to food safety.

## Methodologies for Recovery of *Listeria monocytogenes* and Other *Listeria* species From Environments

Isolation of *L. monocytogenes* from a wide range of environmental samples from food processing plants, farms, or natural environments is challenging due to the numerous background microbiota (e.g., aerobic spore-formers, Enterococci, lactic acid bacteria, coryneform bacteria, *Pseudomonas*, and *Enterobacteriaceae*) that can act as competitors and the environmental factors that cause stress during enrichment (Stessl et al., 2009; Zilelidou et al., 2016). There are several protocols for the detection of *L. monocytogenes* in environmental samples; what they all have in common is the critical step of the enrichment process where the number of *L. monocytogenes* is increased to allow detection. Generally, an efficient enrichment process for the recovery of *L. monocytogenes* from the environment uses a two-phase process with a smaller number of selective additives in the first step. Incubation of *Listeria* enrichment broths at low temperatures has long been common practice (Gray et al., 1948; Gray, 1960), relying on the psychrotrophic nature of *Listeria* to perform selection. Van Renterghem et al. (1991) did not achieve a satisfying recovery of *L. monocytogenes* by cold enrichment techniques. The same was observed by Hayes et al. (1991), who compared the cold enrichment technique with the standardized U.S. Department of Agriculture (USDA) method and realized that the USDA method showed significantly better isolation rates (96 versus 59%). Moreover, some members of the *Listeria sensu lato* group (e.g., *Listeria floridensis*, *Listeria aquatica*, and *L. fleischmanii*) will not grow below 7°C, which can be particularly relevant if a complex consortium of *Listeria sensu stricto* and *Listeria sensu lato* is to be successfully detected (den Bakker et al., 2014; Barre et al., 2016).

A detailed characterization of the media used for *Listeria* enrichment and its evolution can be found in the review by Curtis and Lee (1995).

Currently, the U.S.A. Food and Drug Administration (FDA) Bacteriological Analytical Manual method for the detection of *L. monocytogenes* in food and environmental samples recommends a four-hour pre-enrichment at 30°C in Buffered Listeria enrichment broth (BLEB), followed by the addition of the selective supplements and a further incubation period for 24 to 48 h (Hitchins et al., 2017).

The United States Department of Agriculture (USDA) recommends the use of the media developed at the University of Vermont (UVM) to perform the enrichment. A first enrichment step in UVM-I broth with incubation at 30°C for 20–26 h depending on the sample matrix is recommended, followed by a second enrichment in morpholine propane sulfonic acid buffered *Listeria* enrichment broth (MOPS-BLEB) at 35°C for 18–24 h (USDA, 2019). In the European Union, ISO standards are more frequently used and are the base for legal compliance. According to ISO standard 11,290-1 (ISO 11290-1, 2017), *Listeria* detection is performed using a two-step enrichment procedure: a 24 ± 2 h incubation of the sample in Half-strength Fraser at 30°C is followed by a secondary enrichment in Fraser for 24 ± 2 h at 37°C. A description of the media composition and selective agents as well as the various differential media used for plating and identification can be found in a review by Law et al. (2015).

The impact of the methodology used for enrichment is still a matter of current research as it has been repeatedly seen that the performance of enrichment media is affected by the nature of the sample and greatly influences the time required for *L. monocytogenes* detection (Salazar et al., 2020). Etty et al. (2019) have shown that *Listeria* enrichment broth (LEB) allowed a better growth of *L. monocytogenes*, when compared to Fraser broth and UVM, due to the presence of dextrose that promotes the expression of the p60 protein observed to be proportional to the cellular growth.

The selective enrichment step is further complicated in challenging environments such as soils, where bacterial competition may play a role and lead to the loss of *L. monocytogenes* and other *Listeria* species (Keys et al., 2016; Zilelidou et al., 2016). It has even been observed that certain *L. monocytogenes* genotypes outcompete each other: In selective enrichment UVM, lineage I strains are displaced by lineage II strains (Etty et al., 2019). Zilelidou et al. (2016) using a limited number of strains, described a strain-specific dominance in the ISO protocol; however, no correlation to lineages was established. No serotype nor lineage bias was observed in buffered *Listeria* enrichment broth (BLEB) in a study by Gorski et al. (2006).

Keys et al. (2016) obtained the highest *Listeria* populations in selective enrichments by adding sodium chloride and lithium chloride at higher temperatures (35°C versus 30°C). The authors pointed out the limited number of selective substances for use in *Listeria*-specific enrichment media and thus resulting in only limited selective formulations. There is a need for optimization of *Listeria* selective enrichments to improve recovery from the increasing diversity of *Listeria* species, especially in samples with a high baseline microbial load.

A recent publication by Carlin et al. (2021c) has looked into the performance of enrichment broths used in reference methods (FDA, USDA, and ISO) given the increased number of *Listeria* species described in the last years. This study expanded on the work by Barre et al. (2016) increasing the number of different species and evaluating the USDA and FDA broth on top of the ISO standard as well as three selective and differential agars. The authors observed a weak ability to recover new *sensu lato Listeria* species for the ISO, and both for ISO and USDA enrichment procedures, the growth of other *sensu* lato *Listeria* species could lead to the outgrowth of two *sensu stricto Listeria* species. Only, the BLEB broth used in the FDA method was capable of recovering all 19 species tested by Carlin et al. (2021c). These authors also observed atypical colony morphologies and growth inhibition on selective and differential agars used in the three studied methods for both *sensu stricto* and *sensu lato* species highlighting the potential for false negative results, hence recommending the inclusion of a comprehensive *Listeria* species panel for method evaluations (Carlin et al., 2021c). These results can have a large impact in the adequate detection of *Listeria* and therefore prompt the need for an exhaustive and inter-laboratorial study for following the growth of standardized strains, representative of all the different species, throughout each of the steps of the different methods.

The direct application of molecular methods has also been widely explored. However, Locatelli et al. (2013) showed that the sole application of molecular detection methods after direct DNA extraction from soil samples underestimated the presence of *L. monocytogenes* in soil compared to the cultivation-based method. Further characterization of *Listeria* isolates, including species differentiation, was often performed by *sigB* sequencing for allele typing in combination with multiplex PCR (Doumith et al., 2004; Nightingale et al., 2007).

New approaches combining quasimetagenomic approaches with microbiome analyses based on 16 S RNA sequencing provide good insights into the co-selection of *L. monocytogenes* in the presence of a diverse background flora (e.g., *Serratia*, *Pseudomonas*, and *Enterococcus*, *Bacillus*) during selective enrichment. Wagner et al. (2021) tended to use a shortened enrichment time of 4 h for molecular detection to fully capture *L. monocytogenes* genotype diversity based on molecular methods.

In source tracking approaches in contaminated ice cream applying the latter novel tools, Ottesen et al. (2020) showed that *L. monocytogenes* was inhibited by the competitive flora (*Anoxybacillus* and *Geobacillus*) until 20 h of incubation. The number of species decreased with increasing the enrichment time, and *L. monocytogenes* and *Rothia mucilaginosa* were the only species detected at all-time points (Commichaux et al., 2021).

## The Prevalence of *Listeria* in the Environment

As with some other Gram-positive microorganisms, such as spore-formers, the occurrence of *Listeria* species is related to habitat, such as water, soil, and decaying vegetation (Sauders and Wiedmann, 2007), although prevalence remains variable. As stated before, one of the factors for this variability is attributed to the use of different enrichment protocols, but the wide range of environmental parameters, such as climatic conditions and soil composition, also determine the survival of a strain. The microbiota, mesobiota, and macrobiota present in the environment were observed to be critical. Vivant et al. (2013) compiled relevant cases of the occurrence of *L. monocytogenes* between 1971 and 2013. The authors reviewed the literature on the influence of the soil characteristics in the population dynamics and conclude that the short-term survival variability can be attributed to the soil chemical properties, such as the basic cation saturation ratio, whereas the long-term survival variability is attributed to the soil texture, namely, clay content. The pH of the soil is a major factor influencing the survival of *L. monocytogenes* in soil. Table 1 updates the review by Vivant et al. (2013) covering the period of years 2002 to 2020. Prevalence data in these studies indicate *L. monocytogenes* presence at the limit of detection and up 19% in soil and between 8.9 and 43% in water. In a nationwide survey in the United States of America, a 31% *Listeria* prevalence was detected out of 1,004 investigated soil samples, with 594 *Listeria* spp. isolates with different *sigB* allele types. Variables related to soil properties, climate, and land use were evaluated, and salinity, molybdenum concentration, and moisture were identified as the most important variables in explaining the presence or absence of *Listeria* (Liao et al., 2021).

**Table 1:** *Listeria* spp. occurrence in the natural environment (2002–2020).

Sample type	Occurrence (%)	Country	Year	Reference
**Solid material**				
Soil/environment	19.0**	USA	2001–2002	Sauders et al., 2012
Soil	17*	France	2011	Locatelli et al., 2013
Soil/agriculture	16.0*	USA	2009–2011	Sauders et al., 2012
Soil/farm environment	14.6*	USA	2001–2003	Nightingale et al., 2004
Soil/agriculture	5.4*	India	2003	Moshtaghi et al., 2003
Soil/farm environment	3*	Ireland	2009	Fox et al., 2009
Soil (wilderness)	0*, 4.44**	USA	2009–2010	Ahlstrom et al., 2018
**Liquids**				
Surface water (watershed)	30.1*	USA	2012	Gorski et al., 2014
Surface water	10.0*	Canada	2005	Lyautey et al., 2007
Water (rural environment)	8.9*	Iran	2014	Nassirabady et al., 2015
Water (urban environment)	15.2*	Iran	2014	Nassirabady et al., 2015
Surface water (watershed)	43*	USA	2014	Cooley et al., 2014
Water (wilderness)	1.11*, 5.56**	USA	2009–2010	Ahlstrom et al., 2018
**Mixed sample types**				
Soil, water and vegetables	16.58*, 78.24**	Nigeria	n. s.	Oyinloye et al., 2018
Soil, water	14*	Bangladesh	n. s.	Manjur et al., 2018
Soil/ irrigation water	14*	South Africa	n. s.	Iwu and Okoh, 2020

* Occurrence of *L. monocytogenes*;

** Occurrence of *Listeria* spp.

Chapin et al. (2014) studied the ecology of *Listeria* and found that in the case of *L. monocytogenes*, prevalence was not only related to seasons and geographic factors, but that meteorological factors and proximity to water and pastures also played an important role. By analyzing five sampling sites in New York State, the author described a prevalence of *L. monocytogenes* in natural and agricultural environment that ranged from 8.0% (59/734) to 15.0% (88/588). The prevalence of *Listeria* spp. was significantly higher in summer (37%) than in fall and spring in production areas.

It has been hypothesized that the ability of the genus to adapt to cold environments would contribute to a higher detection at high altitudes. However, Linke et al. (2014) observed that the overall prevalence of *Listeria* spp. at altitudes above 1,500 m was very low (2.6%) and the few isolation events were +shown to originate from members of the *L. ivanovii* and *L. seeligeri* species. Factors other than higher elevation, such as UV radiation, intense freeze–thaw cycles, a shorter growing season, and lower human and animal densities with consequently less runoff bringing *Listeria* spp. into the soil, created harsher environmental conditions (Lyautey et al., 2007; Strawn et al., 2013; Cooley et al., 2014). The latter research on natural reservoirs of *Listeria* species in environmental ecosystems also confirmed the importance of proximity to water sources by finding higher isolation rates of *L. monocytogenes* and *L. innocua* in samples collected from floodplains of the Danube and Schwarza rivers in Austria (Linke et al., 2014).

The presence of water was also identified as a relevant risk factor in soil samples by Weller et al. (2015b). The authors observed that, in field crops environments, among other factors, the period between irrigation and sampling was statistically significant for detection probability, with 3 days having an odds ratio of about 39 for *L. monocytogenes* and about 5 for *Listeria* spp., respectively, compared to a period of 10 days or more. However, Castro-Ibáñez et al. (2015) found that, unlike other foodborne pathogens, the time following a flooding event did not affect the risk of *L. monocytogenes* contamination in lettuce fields and hypothesized that this was due to the low level of contamination with this pathogen in floodwater.

Isolation of the various *Listeria* species differed significantly between natural and urban environments, with *L. seeligeri* and *L. welshimeri* being more abundant in the former and *L. monocytogenes* and *L. innocua* in the latter (Sauders et al., 2012).

These differences have been explained by factors such as fecal excretion of *Listeria* spp. by wildlife or soil properties that favor the survival of certain species (Sauders and Wiedmann, 2007; Hellström et al., 2008). The ability of different *Listeria* species to utilize different carbon sources also plays a role. The fact that *L. ivanovii* and *L. seeligeri* are able to utilize xylose from the decomposition of cellulose may allow these species to survive longer in soil where this nutrient is abundant (Sauders and Wiedmann, 2007). Nevertheless, the association between *Listeria* and soil was noted in a study conducted in urban areas, where the highest prevalence was found in samples taken from treaded shoe soles rather than smooth treaded soles (12.5–20.7% vs. 0–6.6%), and near urban parks rather than shopping malls (Schoder et al., 2015).

## *Listeria monocytogenes* Genotypes Prevalence in the Environment

Various typing systems are available for genetic characterization of *L. monocytogenes*. Serotyping, based on somatic and flagellar antigen differences, distinguishes 14 serotypes (Farber and Peterkin, 1991; Yin et al., 2019). Four genetic lineages can be discriminated by sequence-based methods. Pulsed-field gel electrophoresis (PFGE) is a gel-based subtyping method with superior discriminatory power. Multilocus sequence typing (MLST) is a sequence-based method that focuses on the typing of seven housekeeping genes and allows assignment to sequence types and clonal complexes. The most internationally recognized scheme was proposed by Ragon et al. (2008), an adaptation of the initially proposed scheme by Salcedo et al. (2003). This typing method is still currently used (Kaszoni-Rückerl et al., 2020; Stessl et al., 2021).

Most listeriosis outbreaks are caused by serotypes 1/2a, 1/2b, 1/2c, and 4b (Cossart, 2011). Isolates of genetic lineage I are associated with sporadic and epidemic listerioses in humans, whereas isolates of lineage III are more commonly associated with listerioses in animals (Orsi et al., 2011; Buchanan et al., 2017).

**Figure 1:**
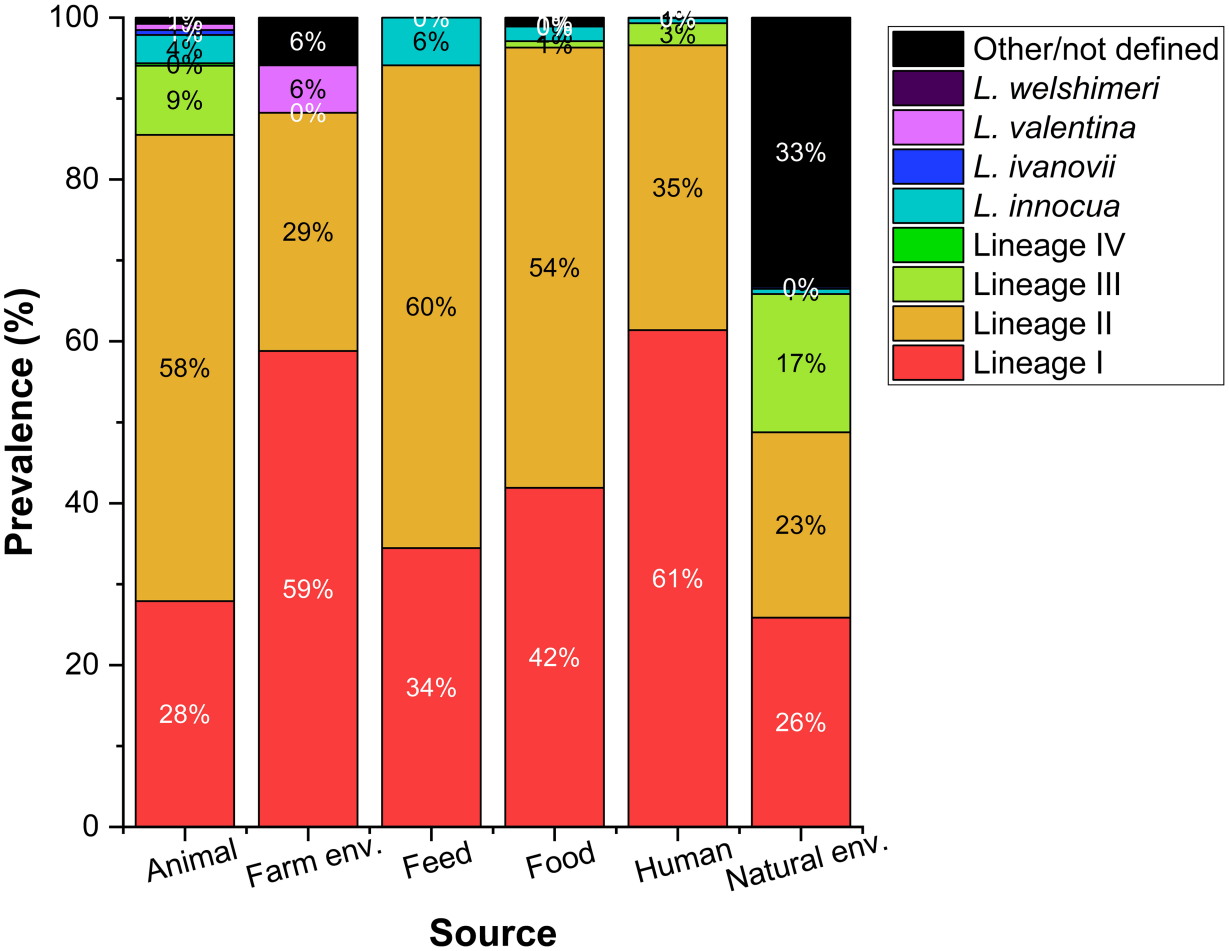
Prevalence reported as percentage of *Listeria* species including *L. monocytogenes* genetic lineages (I–IV) according to different sources. Data collected from the BIGSdb-Listeria (Moura et al., 2016) on August 18th 2021 for a total of 656, 17, 119, 1,105, 1,435, and 773 isolates from each source.

*L. monocytogenes* genetic lineage I and II are the most prevalent *Listeria* MLST profiles in the BIGSdb-Listeria (Moura et al., 2016; Figure 1). Genetic lineage I is more present among human isolates, and lineage II is often assigned to the food and environmental niche (Lomonaco et al., 2015). However, when analyzing this type of data, it is important to bear in mind that the database may be biased as its representativeness is unknown.

Recombination does not occur uniformly throughout the *Listeria* genome, nor across genetic lineages. den Bakker et al. (2008) identified a higher recombination rate in genetic lineage II, more successful in the adaption to different environments and the survival of stress factors, than lineage I. These authors tested for point mutations and recombination using the 7-locus MLST scheme and found that the occurrence of recombination was about six times more frequent in lineage II than in lineage I, when compared to the occurrence of point mutations. These differences between lineages contribute to the differential detection of lineages I and II in environmental isolates. Linke et al. (2014) and Stea et al. (2015) found a nearly identical distribution of these genetic lineages in samples collected in Austria and Canada, respectively.

Zamudio et al. (2020) compared food and human isolates from Switzerland, Germany, and Netherlands with a total of 708 genomes using a cgMLST scheme including 1701 genes (Ruppitsch et al., 2015; Halbedel et al., 2018). These authors observed that sublineage allelic diversity within lineages II and I was greater using a cgMLST approach within lineage I, than within lineage II. However, when this analysis was performed considering SNPs differences within strains, genetic lineage I had a lower genetic diversity (with about 5,000 SNPs difference), while genetic lineage II had about 15,000 SNPs difference.

**Figure 2:**
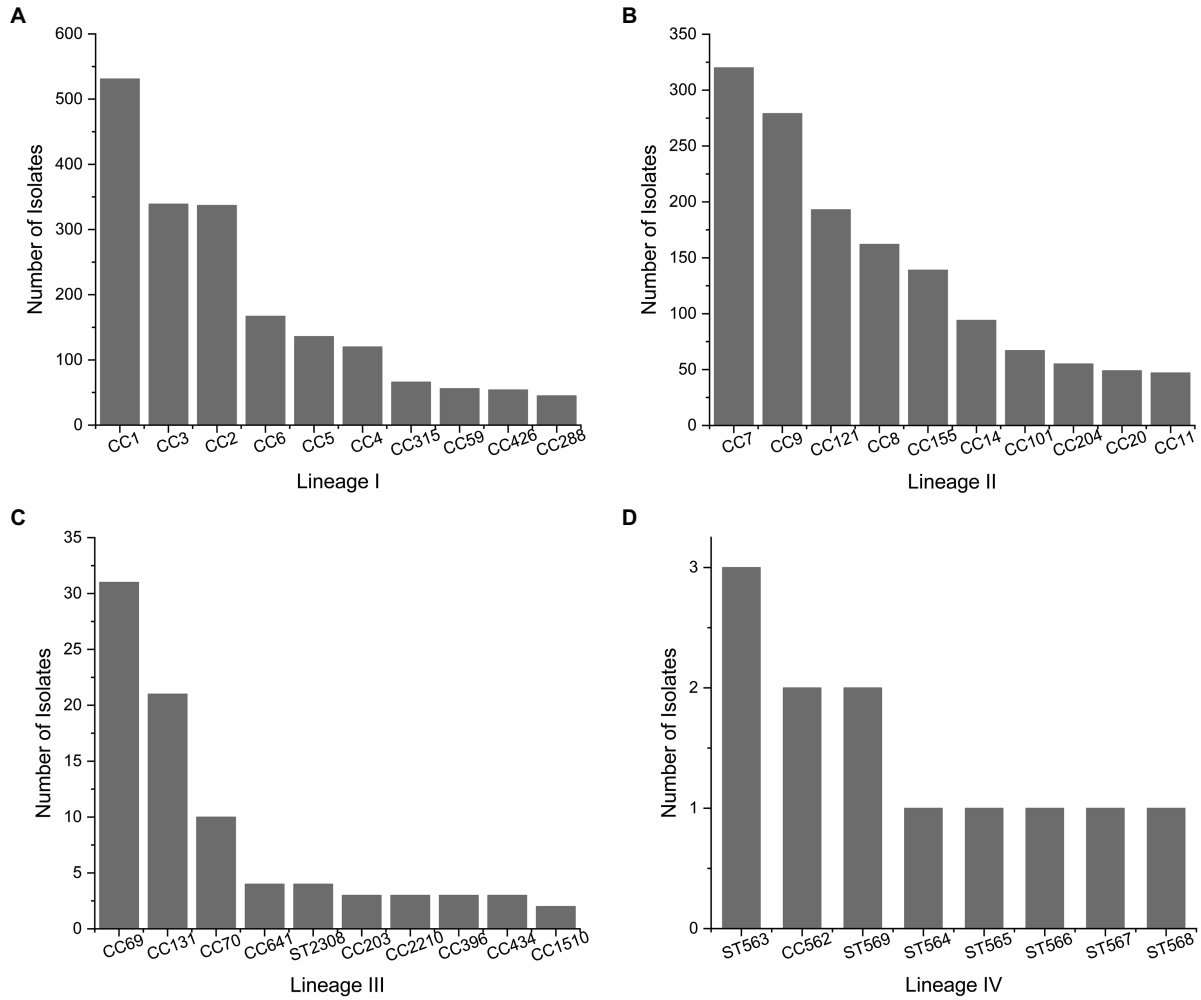
Most Abundant clonal complexes of *L. monocytogenes* for genetic lineages. **(A)** Lineage I, **(B)** lineage II, **(C)** lineage III, and **(D)** lineage IV. Data collected from the BIGSdb-Listeria (Moura et al., 2016) on August 18th 2021 for a total of 2087, 2025, 283, and 12 isolates in lineages I, II, III, and IV, respectively.

The most abundant *L. monocytogenes* clonal complexes (CCs) assigned to each genetic lineage according to the BIGSdb-Listeria (Moura et al., 2016) on August 18th 2021 are shown in Figure 2. We draw the reader’s attention to the unknown representativeness of data included in publicly available databases as analyzed in Figure 1.

The distribution of *L. monocytogenes* in certain areas is influenced by the progressive expansion of genotypes harboring plasmids, transposons, prophages, and *Listeria* genomic islands (e.g., LGI2 for arsenic resistance; Lee et al., 2017; Muhterem-Uyar et al., 2018). Heavy metal resistance is closely linked to the ability of *L. monocytogenes* to cause disease, especially in 4b isolates. CC2 and CC1 harbor the highest prevalence of arsenic resistant isolates, followed by CC4, CC315, and CC9.

Most CC8 isolates contain *Listeria* genomic island 1, which contains the *emrE* gene and increases tolerance to quaternary ammonium compounds (QACs; Kovacevic et al., 2016). Certain ST121 harbor the transposon *Tn*6188, which is also involved in sublethal adaptation to QACs (Müller et al., 2013). Some key genetic features of *L. monocytogenes* are conserved in certain genotypes, such as stress survival islet 1 (SSI-1) and SSI-2, which enhance adaptation to acidic/bile and alkaline/oxidative stress, respectively (Ryan et al., 2010; Harter et al., 2017). The presence of SSI-1 is clonal among *L. monocytogenes* CCs 3, 5, 88, 224, and 315 (genetic lineage I) and CC7, 8, 9, 155, 193, 199, 204, and 321 (genetic lineage II; Hingston et al., 2017; Malekmohammadi et al., 2017). Clonality has also been observed for SSI-2, which is present in *L. monocytogenes* CC121 and as a genetic insert common in *L. innocua* strains (Hein et al., 2011).

Current genomic studies of *Listeria sensu stricto* and senu lato are enriching knowledge of the diversity and adaptability of phylotypes and genotypes, and some inferences can be made about *L. monocytogenes*. In fact, Liao et al. (2021) compiled a US-wide atlas of 1854 *Listeria* isolated from soil and determined through whole-genome analysis that the “cosmopolitan” phylotypes had a more open pangenome and were affected by positive selection, which was reflected in their core genome.

## Molecular Determinants of *Listeria monocytogenes* Saprophytic Lifestyle

*L. monocytogenes* can survive and multiply in the complex and diverse environment of soil. Soil is usually composed of organic matter, plant roots, decaying vegetation, minerals, and a variety of microorganisms and invertebrates. The availability of nutrients, water, pH, and interactions among organisms living in the soil determine gene expression. The importance of soil and water sources to the genus *Listeria* is reinforced by the fact that newly described *Listeria* species and subspecies were often associated with these niches (Carlin et al., 2021a).

In general, sigma factors play an important role in metabolism and the transition to a pathogenic lifestyle. In particular, the SigB regulon (σ^B^) has been identified as an important regulon controlling several functions, including stress response and virulence (Dorey et al., 2019). A recent systematic literature review on this topic identified 304 genes with σ^B^-dependent consensus promoters encoding various functions, including stress response (e.g., osmotic, oxidative, and acid stress; *n* = 73) and virulence (*n* = 51; Liu et al., 2019).

The SigB controlled stress response mechanisms facilitates the adaptation of *L. monocytogenes* to the soil environment. Using a sigB deletion mutant, Gorski et al. (2011) observed the role of SigB, in the initial adaptation phase as well as long-term survival in soil associated with transition to and survival in stationary phase. It has been clearly demonstrated that SigB regulation is essential for the survival of *L. monocytogenes* in nature, although the factors activated by SigB under these severe conditions remain to be deciphered (Dorey et al., 2019).

The accessory gene regulator (Agr) system is an auto-inducible transcriptional regulator associated with virulence as well as biofilm formation. The Agr system is organized as a four-gene operon, *agrBDCA*. By using deletion mutants on *agrA* (response-negative mutant) or the signal pro-peptide AgrD (signal-negative mutant), Vivant et al. (2014b) demonstrated that the Agr communication system is required for optimal survival of *L. monocytogenes* in soil, especially when complex microbial communities are active. The transcriptome of an *agrA* deletion mutant also differs significantly from the wild type under soil conditions, in contrast to abiotic conditions. Vivant et al. (2014a) observed that 578 protein-coding genes related to the cell envelope and cellular processes, as well as several non-coding RNAs, were differentially transcribed. In addition, the Agr system positively regulates the chitinase expression (Paspaliari et al., 2014). Transcriptional microarray analysis during growth of *Listeria* in soil extracts revealed that other enzymes involved in catabolism and nutrient acquisition mechanisms also exhibited higher levels of transcription to respond to soil nutrient scarcity and to allow utilization of abundant carbon sources such as cellulose or chitin (Piveteau et al., 2011).

The SigB and Agr systems act synergistically, as the ability to colonize grass roots under sterile soil conditions was significantly impaired in the double mutant compared to the wild type and each of the single mutants. Under biotic conditions, strains viability decreased over time for both wild type and mutants, although this effect was stronger in the double mutant (Marinho et al., 2020).

Brunhede et al. (2020) demonstrated that the LisRK two-component system is important for growth in sterile soils and survival in non-sterile soils. The authors observed reduced fitness in two different soil types (silty clay and loamy sandy soil) of isogenic mutants of *lisR* and *lisK* compared to the wild-type strain. Loamy sandy soils showed a greater decrease in bacterial population when the experiment was conducted in non-sterile soil. A mutant lacking all seven sRNA-coding genes of the *lhrC* family was used to show that it is involved in the regulation of *L. monocytogenes* gene expression in soil. While residing as a saprophyte in the environment, *L. monocytogenes* may be uptaken by susceptible hosts and become an intracellular pathogen.

The key transcriptional regulators PrfA and SigB play a central role in mediating the switch between these two lifestyles. Detailed information on the interaction between these regulators can be found in a recent report by Gaballa et al. (2019).

Vivant et al. (2017) studied the changes in the transcriptome of *L. monocytogenes* CIP 110868 when adapted to lagoon effluents and soil. Genes involved in mobility, chemotaxis, carbohydrate transport, and the cell envelope were upregulated, especially in lagoon effluents.

Tan et al. (2016) studied the importance of L. *monocytogenes* attachment to plant cell walls and identified the role of pectin and xyloglucan important for the attachment process. Overall, the attachment was dependent on strain-specific properties but not directly influenced by the strain concentration.

Natural environments can be a reservoir for particularly virulent strains. Internalin A (InlA), which plays an important role in *L. monocytogenes* virulence, is intact in 90% of isolates from natural environments (Gorski et al., 2016), in contrast to isolates from food processing, where approximately 35% of isolates have a truncated *InlA* gene (Kovacevic et al., 2013). In food, processing environments and food *L. monocytogenes* sublineages (SLs) dominantly present are SL9, SL121, among others (SL101 and SL5), which harbor genes relevant for Benzalkonium chloride tolerance. Interestingly, 31% *L. monocytogenes* isolates indicated truncated *inlA* genes (Hurley et al., 2019).

Linke et al. (2014) observed in a subset of 16 isolates from soil that about two-thirds of the isolates had a truncated *inlA* gene, while one-third had an intact *inlA* gene. This is particularly relevant to adaptation to natural environmental stresses, as Hingston et al. (2017) observed that when *L. monocytogenes* with a full-length *inlA* gene were compared to those with a premature stop codon, the former was found to have significantly improved cold tolerance.

Raschle et al. (2021) reported hypervirulent *L. monocytogenes* CC1, CC4, CC6, and CC412 in river ecosystems. Therefore, the latter *L. monocytogenes* genotypes with intact *inlA/B* and *prfA* genes and LIP-4 (CC4) also have a reservoir in surface waters, which should be considered as a risk for transmission to humans and animals.

## Antimicrobial Tolerance and Resistance in environmental *Listeria monocytogenes*

Currently, there are informative publications that critically illuminate the subject of *L. monocytogenes* and the tolerance or potential resistance to antimicrobials (Yin et al., 2019; Baquero et al., 2020). There are some papers that have focused on antimicrobial resistance in *L. monocytogenes* in the past and have used the term resistance without EUCAST or CLSI clinical breakpoints being available for each of these test substances. In the case of *L. monocytogenes*, the use of the term “increased tolerance” when observing increased MIC levels to a test substance without existing clinical breakpoints is certainly more appropriate. According to EUCAST, clinical breakpoints are available for penicillins, meropenem, erythromycin, and trimethoprim-sulfamethoxazole.^1^ Some authors expand the range of clinical breakpoints with the available ones of enterococci or staphylococci (Conter et al., 2009; Bertsch et al., 2014).

The number of antimicrobials to which *L. monocytogenes* was more tolerant or even resistant in literature were tetracycline, clindamycin, ampicillin, and erythromycin (Supplementary Table S1). The percentage of tolerant *L. monocytogenes* strains to antimicrobials varies among studies, but in some studies, all test strains were more tolerant to at least one antimicrobial agent.

*L. monocytogenes* is usually susceptible to penicillins, trimethoprim, aminoglycosides, macrolides, and vancomycin (Troxler et al., 2000; Bertsch et al., 2014), but most strains show native resistance to cephalosporins, first generation quinolones (nalidixic acid and cinoxacin), sulfamethoxazole, fosfomycin, oxacillin, and lincosamides (Olaimat et al., 2018).

The majority (88.5%) of *L. monocytogenes* isolated in a study by Linke et al. (2014) indicated a higher minimum inhibitory concentration (MIC) to cefotaxime and 35% of isolates to ceftriaxone. These results are also consistent with those of Troxler et al. (2000) who described a natural resistance of most *Listeria* strains to modern cephalosporins. Soni et al. (2014) also observed a higher tolerance to cefoxitin, a second-generation cephalosporin. In addition, 60% of the soil isolates were more tolerant to ciprofloxacin.

*L. monocytogenes* isolated from soil, water, and vegetables in Nigeria were reported to be resistant to the β-lactam group (ampicillin and amoxicillin) and to more than one antibiotic class (Oyinloye et al., 2018). Manjur et al. (2018) found a high percentage of strains resistant to ampicillin and penicillin (71%) and 100% resistance to erythromycin in urban soil samples. Recent articles recommend retesting the strain panel, because ampicillin resistance occurs in only a small percentage of isolates (<1%), due to possible horizontal gene transfer (HGT) of *E. faecium* (Baquero et al., 2020).

In some studies, certain *L. monocytogens* sequence types (STs) indicated increased tolerance or resistance to antibiotics: Yan et al. (2019) reported antimicrobial resistance (tetracycline, erythromycin, chloramphenicol, and trimethoprim-sulfamethoxazole) in ST3, ST8, ST9, ST155, and ST515 food isolates. The predominant ST9 indicated resistance acquired by horizontal gene transfer. Certain genotypes present in soil samples (ST1, ST2, ST6, ST7, ST20, ST37, and ST91) showed an increased tolerance to cephalosporine. ST7, ST20, ST2, and ST6 were more tolerant to erythromycin and ST20 to linezolid (Linke et al., 2014).

Antimicrobial increased tolerance or even resistance in *L. monocytogenes* can be caused by efflux pumps or the acquisition of genetic elements, like plasmids and transposons (Matereke and Okoh, 2020). Some of the resistances, such as those against fosfomycin, depend on PrfA-dependent cell uptake mechanisms and may only occur under lab conditions, because PrfA is downregulated when nutrients such as phosphorylated sugars are available (Scortti et al., 2006).

Kremer et al. (2017) described a plasmid and efflux transporter in *L. monocytogenes* that lead to benzalkonium chloride tolerance in combination with higher MICs for gentamicin and amoxicillin. Since benzalkonium chloride is used for disinfection in food processing plants, co-selection of resistance mechanisms could lead to difficulties in listeriosis treatment. Oliveira et al. (2017) described that *L. monocytogenes* strains adapted to subinhibitory concentrations of resveratrol, a natural product used as a food preservative, were more tolerant to heat and acid stress.

Strains exposed to environmental stress may be resistant to higher levels of antibiotics due to stress adaptation (Lungu et al., 2011). Accumulation of heavy metals, such as mercury, cadmium zinc, arsenic, copper, and lead in the environment, leads to co-selection of antibiotic resistance in Gram-positive bacteria. Metal-related antibiotic-resistant determinants that reside on the same mobile genetic elements can be horizontally transferred from the bacteria in which they evolved (e.g., staphylococci, enterococci, and lactic acid bacteria) to related pathogens such as *L. monocytogenes* (Imran et al., 2019; Parsons et al., 2020).

Recently, microplastics polluting the marine environment were found to harbor metals, antibiotics, and pathogenic bacteria and provide a favorable environment for metal-induced co-selection of multidrug-resistant pathogens (Imran et al., 2019). Zhao et al. (2019) investigated antibiotic resistance genes (ARGs) in metal-contaminated urban and suburban soils collected in the western part of Northern Ireland and detected 164 ARGs. Sample types included stream water and sediment, heavy mineral concentrate, surface, and deep soil (Smyth, 2007). The authors detected an average of 3.4 × 10^7^ ARG gene copies per gram of soil and observed a correlation between the presence of heavy metals in soil and the spread of ARGs through horizontal gene transfer.

This indicates the need to further elucidate the mechanisms behind antimicrobial resistance and cross-resistance and to raise awareness of the impact of the use of preservatives, disinfectants, and environmental contaminants.

## *Listeria* in the Environment and its Entrance Into Food Processing better facilities

Hof (2003) provided an overview of the history and epidemiology of listeriosis and referred to the work of Schlech et al. (1983) as a milestone in listeriosis research, because it established the link between the disease and a contaminated food—coleslaw. Indeed, *Listeria* that occur in the natural environment often find their way into food processing facilities and the food chain through raw materials. Proximity to wildlife, birds and livestock and fecal contamination are closely related to the prevalence of *L. monocytogenes* during crop production. As mentioned earlier, pre-harvest conditions, such as manure application and irrigation water quality, not only determine the prevalence of *Listeria* in produce, but also favor the presence of *Listeria* at levels that are not manageable and controllable with established practices in the industry.

Several crops that were contaminated before harvest have been implicated in foodborne outbreaks, namely, mushrooms, celery, melons, apples, lettuce, and corn (European Food Safety Authority and European Centre for Disease Prevention and Control, 2018; Centers for Disease Control and Prevention, 2021). Increased rainfall or flooding is associated with an increased prevalence of zoonotic pathogens in production environments and also in natural environments (Linke et al., 2014; Weller et al., 2015b). The contamination of fresh vegetables with *L. monocytogenes* could be reduced by minimizing irrigating prior to harvesting. The presence of *L. monocytogenes* on seeds facilitates its entry into the plants during its germination. Therefore, seed decontamination is a potential promising method to reduce microbial internalization and diffusion in crops (Miceli and Settanni, 2019).

Practices such as sanitizing food and disinfecting food-contact surfaces are necessary to ensure food safety and, if done inadequately, can lead to contamination of produce during subsequent operations such as cutting and slicing (Zhu et al., 2017). Marik et al. (2020) highlight the importance of produce surface topography and its background microbiota of *L. monocytogenes* growth and survival characteristics on intact fruit and vegetable surfaces during postharvest handling.

*Listeria* is ingested not only by wild animals but also by farm animals and excreted asymptomatically without causing clinical signs. In most cases, *L. monocytogenes* can be isolated from the intestines of healthy deer and wild boar (Weindl et al., 2016) but also cattle, sheep, and pigs (Iida et al., 1991; Hurtado et al., 2017). Palacios-Gorba et al. (2021) monitored the presence of *Listeria* in 19 dairy farms during seven consecutive seasons from autumn 2018 to spring 2020 in cattle, goat, and sheep farms. The authors detected the presence of *Listeria* in 18 dairy farms with *L. monocytogenes* being the second most prevalent species after *L. innocua*. Moreover, the authors observed that within *L. monocytogenes* 70% of the isolates belonged to lineages I. The authors observed as the most prevalent SLs and CCs for animal samples SL1/CC1, SL219/CC4, SL26/CC26, and SL87/CC87 and for the environmental samples SL666/CC666. Therefore, *Listeria* can be shed from farm animals in the agricultural environment and introduced into the food-processing environment if adequate hygiene barriers are not in place. Food processing plant employees can be a source of contamination of the food processing environment due to inadequate hygiene practices or lack of or inadequate protective equipment (Stessl et al., 2020). However, practices in the industry, in animal harvesting and processing plants, are particularly delicate. More than the apparent cleanliness of the animal’s hide, the processes of skinning and evisceration are crucial to the contamination of a carcass with soil and fecal matter, which can then lead to permanent contamination of the processing environment (Demaître et al., 2021).

**Figure 3:**
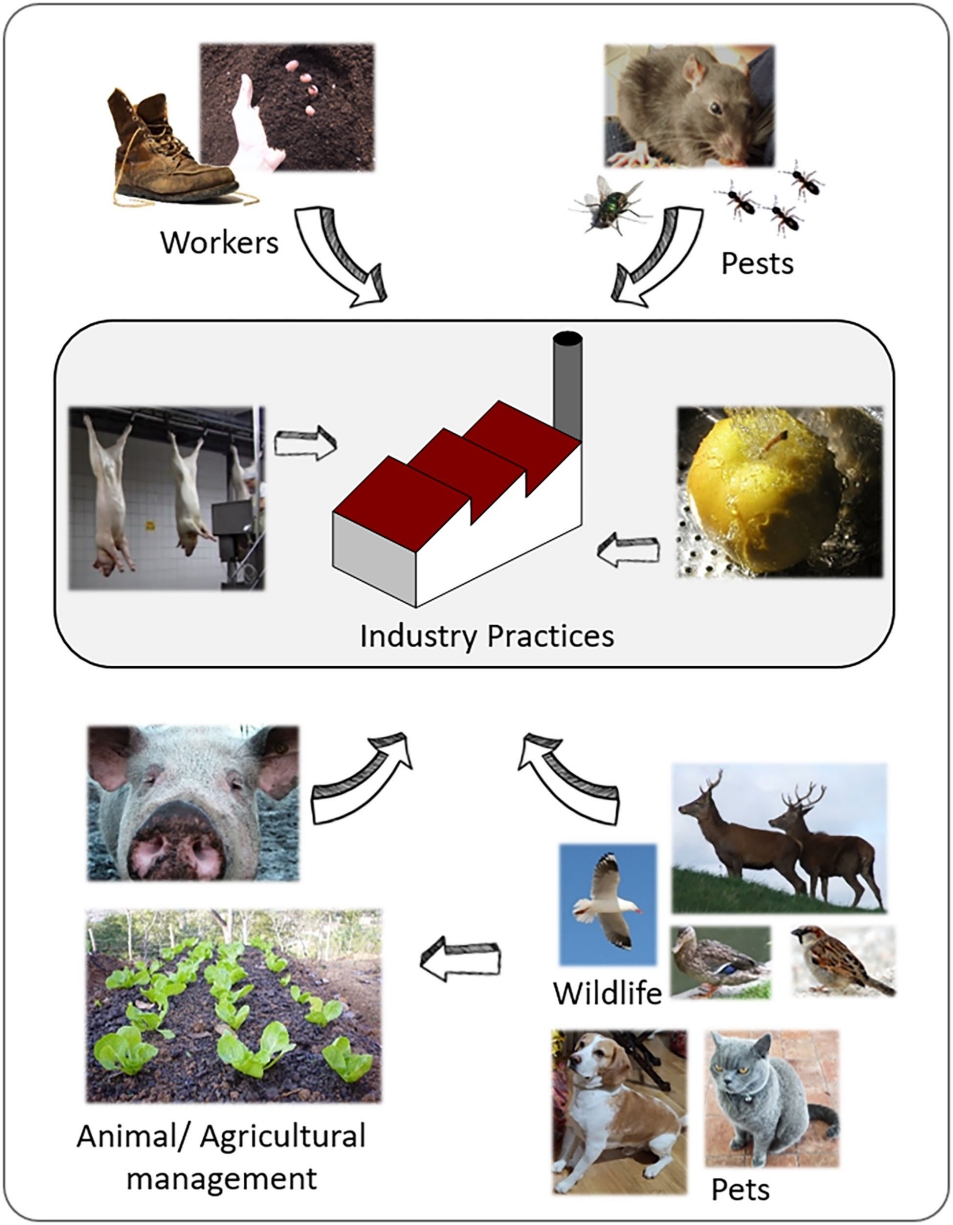
Common routs of entry for *Listeria* in food processing facilities.

Many biotic vectors can play an important role in the transmission of *Listeria* between the environment and food processing facilities, either through direct interaction with the food or animals or as a direct source in a food processing environment. Rodents and insects such as flies and cockroaches have been shown to be a source of *Listeria* (Wang et al., 2017; Li et al., 2018). Wang et al. (2017) observed that *Listeria*, especially *L. monocytogenes* ST87, is present at low levels in the intestinal tracts of wild rodents in the natural environment of China. This is highly relevant as ST87 is one of the most common STs present in the food chain and human listeriosis in China (Li et al., 2018). Figure 3 summarizes the most common routs of entry of environmental *Listeria* into food processing facilities. In detail, the importance of food handling practices within the food facility, the compliance to good manufacturing practices in terms of worker hygiene and pest control, and the role *L. monocytogenes* transmission by wild and domestic animals. The latter might contaminate raw material or food processing environment. Furthermore, environmental factors (e.g., the quality of irrigation water) influence the produce safety.

## Conclusion

The genus *Listeria* currently comprised by 28 species just and as many other Gram-positive microorganisms its occurrence is associated to the natural environmental habitats such as soil and decaying vegetation. However, Listeria’s prevalence in the saprosphere is greatly dependent on many biotic and abiotic factor. Among those factors, the soil type and its characteristics such as pH and mineral composition are determinant. The proximity to water sources is also extremely relevant for the population dynamics. Other factors such as meteorological factors, geographic, and seasonal variations are referred to as relevant. Many biotic factors related to human and other animal density and vegetation have been suggested as explanations for the presence or absence of *Listeria*. Differences in isolation among the different species are often attributed to differential fecal shedding and nutrient usage ability. Moreover, many are the molecular determinants that condition the fitness to the saprophytic lifestyle. The SigB regulon, the Agr system, and the LisRK system determine the fate of *Listeria* in the environment along with the PrfA regulon, which is determinant for the transition to intracellular pathogen lifestyle.

Exposure to environmental stresses, including heavy metals, is of particular importance because it determines the fate of mobile genetic elements that may be related not only to environmental fitness but also to antibiotic resistance, which is an obvious problem for human and animal health. Within the genus, *L. monocytogenes* is a relevant human pathogen that is frequently transmitted through food and causes sporadic and epidemic listeriosis in high-risk groups. This foodborne pathogen finds its way from the saprosphere into the food chain by a wide range of routs that range from the raw materials that may be naturally contaminated or become contaminated by inadequate procedures, the presence of wildlife, pets or pest naturally carriers, or by bad practices of food processing facilities workers.

Only the full awareness of the factors that determine the presence of Listeria in the natural environment can lead to the improvement of the agronomical and zootechnical practices capable of preventing the entrance of *Listeria* in the primary production. Improving industrial practices, including creating appropriate training for personnel that will effectively prevent the occurrence and selection of this microorganism in a food environment, must also be based on a thorough knowledge of the saprophytic lifestyle of *Listeria*.

## Author Contributions

AL and KL prepared the manuscript, figures, and tables. BS and MW designed the review, raised funds, supervised the work, and revised the manuscript. All authors contributed to the article and approved the submitted version.

## Funding

This work was partly funded by the Austrian Competence Centre for Feed and Food Quality, Safety and Innovation (FFoQSI). The COMET-K1 competence centre FFoQSI is funded by the Austrian ministries BMVIT and BMDW, and the Austrian provinces Niederoesterreich, Upper Austria and Vienna, within the scope of COMET—Competence 301 Centers for Excellent Technologies. The COMET program is handled by the Austrian Research Promotion Agency FFG. AL is funded by the Research Leaders 2025 postdoctoral funding from the European Union’s Horizon 2020 research and innovation program under the Marie Skłodowska-Curie grant agreement number 754380.

## Conflict of Interest

The authors declare that the research was conducted in the absence of any commercial or financial relationships that could be construed as a potential conflict of interest.

## Publisher’s Note

All claims expressed in this article are solely those of the authors and do not necessarily represent those of their affiliated organizations, or those of the publisher, the editors and the reviewers. Any product that may be evaluated in this article, or claim that may be made by its manufacturer, is not guaranteed or endorsed by the publisher.

## Footnotes

^1^ https://www.eucast.org/clinical_breakpoints/

## Supplementary Material

Supplementary Table S1 Percentage of antibiotic tolerance or resistance of *Listeria monocytogenes* mentioned in literature in regards to sample type and geographic origin.
